# Unusual Presentation of Duplex Kidneys: Ureteropelvic Junction Obstruction

**DOI:** 10.1155/2016/7960794

**Published:** 2016-10-18

**Authors:** Cemile Başdaş, Süleyman Çelebi, Seyithan Özaydın, Birgül Karaaslan, Elmas Reyhan Alim, Ünal Güvenç, Serdar Sander

**Affiliations:** ^1^Department of Pediatric Urology, Kanuni Sultan Suleyman Education and Research Hospital, Istanbul, Turkey; ^2^Department of Pediatric Urology, Istanbul University, Medicine Faculty, Istanbul, Turkey; ^3^Department of Pediatric Surgery, Kanuni Sultan Suleyman Education and Research Hospital, Istanbul, Turkey

## Abstract

*Aim*. Ureteropelvic junction obstruction (UPJO) is rarely associated with a duplex collecting system. We review this unusual anomaly in terms of presentation, diagnostic evaluation, and surgical management.* Method*. We retrospectively reviewed the medical records of patients diagnosed with a duplex system with UPJO.* Result*. Sixteen patients (6 girls, 10 boys) with 18 moieties were treated surgically and four patients were treated conservatively. The median age at surgery was two years (range, 2 months to 7 years). The lower pole and upper moiety were affected in 12 and two kidneys, respectively, and both were affected in two patients. The anomaly was right-sided in 12 moieties and left-sided in six. The duplication was incomplete in seven patients and complete in nine. The mean renal pelvis diameter at the time of surgery was 25.6 (range 11–48 mm) mm by USG. The mean renal function of the involved moiety was 28.3% before surgery. Management included pyelopyelostomy or ureteropyelostomy in six moieties, dismembered pyeloplasty in eight moieties, heminephrectomy in four cases, and simultaneous upper heminephrectomy and lower pole ureteropyelostomy in one patient.* Conclusion*. There is no standard approach for these patients and treatment should be individualized according to physical presentation, detailed anatomy, and severity of obstruction.

## 1. Introduction

Duplex collecting systems are the most common anomaly of the urinary system and can be either incomplete or complete [1]. Anomalies like a ureterocele and ectopic ureter mostly affect the upper system, while anomalies like vesicoureteral reflux (VUR) mostly affect the lower system [2, 3]. Although a duplicated collecting system and ureteropelvic junction obstruction (UPJO) are common anomalies in pediatric urological practice, they rarely occur together, comprising 2–7% of upper urinary tract anomalies [4].

The diagnosis and management of UPJO associated with a duplicated collecting system can be difficult because of the high variability in anatomy, degree of obstruction, and clinical factors [5]. Most duplications are incomplete, with the confluence of the ureters localized at some point above the ureteral orifice [1]. These forms of duplication rarely give rise to clinical problems, unlike complete duplication anomalies, which often cause symptoms or impair renal function [6]. Surgical correction can be challenging in cases of incomplete duplication when the lower and upper pole ureters combine proximally and the surgical reconstruction method, such as pyelopyelostomy or pyeloureterostomy, must be chosen carefully [7]. Management is influenced by patient age, frequency of febrile urinary tract infection (UTI), lower or upper pole localization, whether duplication is incomplete or complete, renal unit function, and surgeon's preference [8, 9]. There is no best management strategy or guidelines.

We report our experience with the management of lower and upper pole UPJO with complete and incomplete duplex kidneys, with emphasis on surgical reconstruction techniques.

## 2. Method

We retrospectively reviewed the medical records of patients diagnosed with a duplex system with UPJO. Each case was reviewed for the presenting symptoms, anatomy, patient age and gender, physical examination, urinary tract ultrasonography (USG), dynamic renogram, radiodiagnostic findings, and surgery type. Intravenous urography or retrograde pyelography was performed to delineate the anatomy whenever necessary. Dynamic contrast-enhanced magnetic resonance imaging (MRI) was performed only in recent years to delineate the anatomy and the level of obstruction when all other imaging modalities failed to establish the correct diagnosis. Split renal function was evaluated for the upper and lower poles in a dynamic study.

Indications for surgical intervention were symptomatic obstruction, asymptomatic obstruction with increasing dilatation and decreased function of the affected renal moiety, and failure of conservative management. Surgery was performed using an open technique and followed by USG and scintigraphy.

Follow-up studies consisted of a detailed history focusing on surgical complications, persistent dilatation/obstruction, febrile UTI, and flank pain. Each patient underwent USG of the upper urinary tract at hospital discharge, and 3, 6, and 12 months, and yearly thereafter. A renal scan with a MAG3 dynamic renogram was performed only in patients who presented with symptoms or had persistent or increasing dilatation of the affected renal moiety.

The data were analyzed using SPSS software. Continuous variables are reported as averages and the standard deviation (SD) or medians with interquartile ranges.

## 3. Result

We retrospectively reviewed the charts of 20 patients. Sixteen patients (6 females, 10 males) with 18 moieties were treated surgically at our institution between January 1991 and March 2014 for a duplex system associated with obstructive hydroureteronephrosis due to UPJO. The median age at diagnosis was two years (range, 2 months to 7 years). Twelve of the 16 patients presented with clinical symptoms, such as flank pain and urinary tract infection, while four were asymptomatic. The asymptomatic cases were diagnosed upon USG performed for another disorder, such as penoscrotal hypospadias or bilateral undescended testes (Table 1). Eight of the symptomatic patients were diagnosed with segmental pelvicaliectasis on prenatal USG.

**Figure 1:**
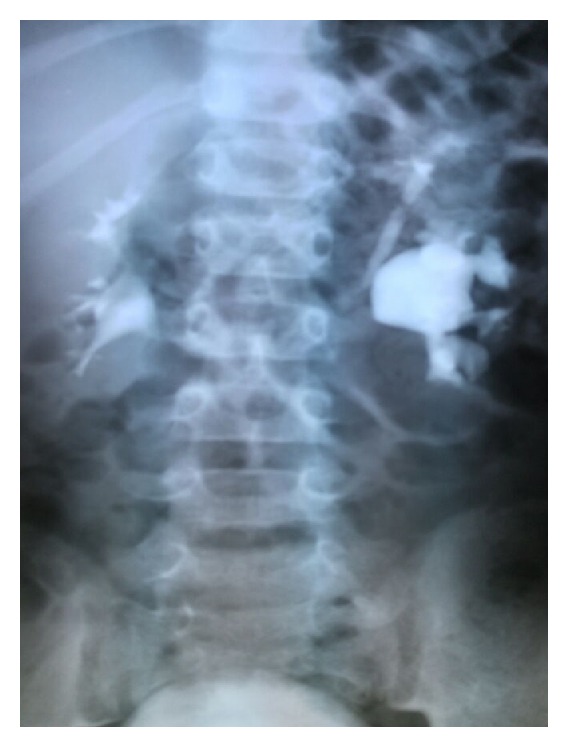
The intravenous urography shows the dilated left lower moiety with an abrupt transition to nondilated ureter at the pelviureteric junction consistent with pelviureteric junction obstruction of the lower moiety. The upper moiety is nondilated.

**Figure 2:**
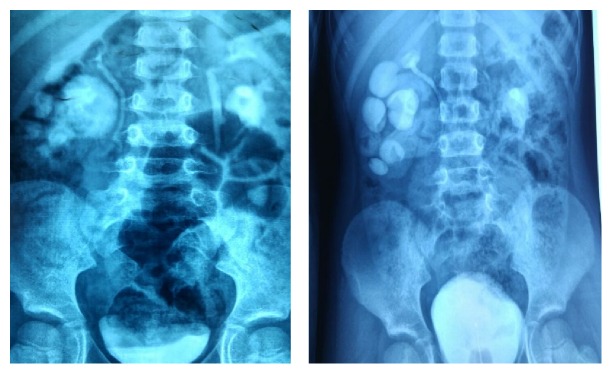
Intravenous examination showing the duplex system on the right side with the UPJO of the lower moiety.

The mean renal pelvis diameter at the time of surgery was 25.6 (range 11–48 mm) mm and the mean function of the involved renal moiety was 28.3% before surgery. MAG3 renal scintigraphy revealed an obstructive pattern or delayed clearance in the involved moiety in all patients. Intravenous urography (Figure 1), retrograde pyelography, and MR urography were performed in 3, 2, and 3 patients, respectively, to delineate the anatomy when all other imaging modalities failed to confirm obstruction of the lower pole (Figure 2).

The lower pole and upper moiety were affected in 12 and two kidneys, respectively, and both systems were affected in two patients. The anomaly was on the right side in 12 moieties and left-sided in six. The duplication was complete in nine patients and incomplete in seven. One patient had a triplex system and one had a multiplex system. One patient had a duplex system on the left side with UPJO of the lower moiety and also had a horseshoe kidney. Associated anomalies included a multicystic dysplastic kidney in the other kidney in two patients, VUR in the other system in two patients, bilateral undescended testes in another two, penoscrotal hypospadias in one patient, and ureterocele in a nonobstructed duplex renal moiety in one patient (Table 1). One patient had a rare anatomic variation with UPJO in the upper system and six elongated infundibula draining the lower system. Concomitant VUR, an ectopic ureter, and a ureterocele were present in one case each and one patient had stone formation in the obstructed system.

Surgery was performed in 16 patients for 18 moieties. This was part of a larger series of 498 patients who underwent surgical therapy for UPJO at our institution during the same period. Thus, the prevalence in our series was 3.2%. The surgery included a pyelopyelostomy or ureteropyelostomy in six patients (6 moieties) with a narrow segment at the ureteropelvic junction (UPJ) of the lower moiety, because the ureteral length between the UPJ and the junction of the lower and upper poles of the ureter was observed during surgery to be insufficient. A dismembered pyeloplasty was performed in eight patients because the length of the lower pole ureter was sufficient and a heminephrectomy was done in four patients with a nonfunctioning cystic system. An upper heminephroureterectomy and lower ureteropyelostomy were performed in a patient with a complete duplex system with lower system obstruction and a nonfunctioning upper system with a blind ureter combined with a ureterocele.

All surgical procedures were performed using an open technique via a flank incision. An internal JJ ureteral stent was used in all patients for 6–8 weeks. In one patient, who required reintervention, the dilatation continued and the function of the renal moiety decreased from 35% to 10%. In all other patients, pelvicaliectasis improved after surgery with no urinary tract symptoms and the anteroposterior diameter of the renal pelvis did not increase. Postoperative uro-MRI and a dimercaptosuccinic acid scan showed equivalent results to the preoperative work-up, with stable renal function and no further impairment.

## 4. Discussion

Renal duplication anomalies are common in the upper urinary tracts of children (0.8%) and are more common in girls [10]. Most are asymptomatic and are discovered incidentally and are seen as a normal functioning kidney with complete or partial duplication [11]. In symptomatic cases, obstruction can occur in the upper pole moiety and can be associated with anomalies like an ectopic ureter or ureterocele, while VUR is associated with the lower pole [12, 13]. UPJO in one moiety in a duplex system is quite rare. Snyder III et al. described four patients with duplex systems in a series of 195 patients with UPJO [14]. Larger series report an incidence of 2–7% of UPJO in duplex systems [15]. In our study, which is one of the largest series to date and focused on surgical cases, the incidence was 3.2% in all cases of UPJO seen in the same period.

The most common cause of antenatal hydronephrosis, which can be either transient or pathological, is UPJO [16]. There are several theories on the causes of UPJO, which may be congenital or acquired and include both intrinsic and extrinsic factors [17]. All of our patients had UPJO due to intrinsic factors with anatomical obstruction confirmed at surgery.

Using current imaging techniques, it is possible to delineate the anatomical and functional status of a duplex system and select appropriate management [18]. USG is a simple method for demonstrating hydronephrosis in obstructed duplicated systems, but it does not allow visualization of the exact path of the ureters [17]. In our series, the diagnosis was correctly established by USG in only 6 of 16 patients. Although we believe that there is no rationale for a routine voiding cystourethrogram, it is sometimes necessary to exclude lower pole reflux when dilated ureters appear on USG. Intravenous urography or pyelography may provide information on the collecting system anatomy. Dynamic renal scintigraphy can show the functional consequences of urinary obstruction, but it has insufficient spatial resolution in cases of reduced renal function. In our department, a dynamic MAG3 renogram is performed in all patients with upper tract dilatation, including asymptomatic patients with renal pelvic dilatation [19]. Uro-MRI may be required in a minority of patients to clarify the anatomy and obtain precise data on ureteral insertion and the degree of obstruction [20]. This imaging technique has been performed only recently in our center and in only five of the patients in our series; it helped to demonstrate the anatomical relationship of the two ureters.

Intraoperative cystoscopy combined with a retrograde ureteropyelogram before surgical correction is another option to clarify the anatomy of duplication anomalies [21]. Three patients required an excretory urogram. In another three patients, the correct anatomy was verified only during surgery.

In duplex systems, the lower moieties are more likely to be subject to UPJO compared with the upper pole [22]. It was observed that the renal function of the lower system with UPJO was good; in the opposite situation, the upper moiety underwent a more rapid loss of function. This may be explained by the fact that the lower segment is the anatomical analogue of a single renal system and usually comprises about two-thirds of the parenchyma, at least two calyces, and a true renal pelvis [23]. In comparison, the upper pole usually has a single infundibulum without a true renal pelvis and has relatively less parenchymal tissue. Therefore, it is possible that the upper pole is exposed to the effects of back pressure. In a recent study, 8 (73%) of the 11 cases had UPJO at the lower pole ureters, while 3 (27%) patients had UPJO at the upper pole ureters [23]. In our series, UPJO was identified at the lower pole in 75% of our patients. Typically, reconstruction was performed for UPJO of the lower poles, while heminephrectomy was usually performed for the upper poles.

In complete duplication, two separate ureteral orifices open into the bladder, while in partial duplication the two ureters fuse before entering the bladder via a single ureteral orifice. Summarizing the reported findings in other cases of UPJO in duplicated collecting systems, the duplication was complete in 55%, incomplete in 39%, and undetermined in 6% [23]. In our series, 56% of the duplications were complete and 44% were incomplete.

Notably, not all patients with UPJO in duplex kidneys need surgical correction as in primary UPJO cases. The obstructive uropathy due to UPJO may be temporary, although some do not improve and others worsen [24]. In a recent study, 33% of the patients were treated conservatively [24]. In our study, 80% of the UPJO patients with a duplex system were managed surgically. The rate varies between 60 and 90% in other series [25].

Indications for surgery include symptoms, obstruction or impaired function of the affected renal moiety, and failure of conservative management. The surgical approach depends on the anatomical features and degree of renal function [26]. Incomplete ureteral duplication can pose a surgical technical challenge when the junction of the upper and lower pole ureters is located proximally and in close proximity to the UPJO [23]. If there is massive hydronephrosis and a dysplastic pole with no or poor function in the parenchyma, a heminephrectomy of the affected pole may be selected [7]. A standard dismembered pyeloplasty is the most appropriate option for complete or nearly complete duplex systems [23]. The length of the lower pole ureter is the major determinant of the surgical technique and mandates individualized surgical treatment. Shelfo et al. reported that the end-to-side pyeloureterostomy was successful and had few complications when the upper and lower pole ureters are located proximally [27]. In our series, the main surgical approach was a dismembered pyeloplasty if the ureter duplication was complete or incomplete but combined at the distal ureter. In cases with an incomplete bifid pelvis or two ureters interacting proximally, we performed a pyelopyelostomy and removed the septum between the pelvises or a ureteropyelostomy was performed if the two ureters could be separated from a proximal level.

An ectopic kidney is a challenge for the pediatric surgeon performing reconstructive surgery [28]. One of our patients had an ectopic duplex kidney with UPJO at the lower moiety and we performed a pyeloureterostomy. One patient had a rare anatomic variant with UPJO in the upper system and six elongated infundibula draining the pelvis in the lower system. This is called a “bagpipe kidney” in our country.

Logically, in a duplex system, UPJO could result from a ureter with a pelvic entry anomaly, such as high insertion or kinking of the UPJ [28]. Nevertheless, in our series intrinsic anatomical obstruction was the cause of the UPJO in all cases. While in standard duplex systems, ureteroceles at the upper lobes or VUR at the lower lobes are common, these were observed in only three of the duplex systems with UPJO in this series; the cause for this is unknown.

## 5. Conclusion

Duplicated collecting systems and UPJO are common anomalies of the urinary system that represent a challenge to pediatric urologists and nephrologists in terms of diagnostic evaluation and type of surgery, despite progress in pediatric radiological imaging, diagnosis, and management. Treatment should be individualized based on clinical presentation, anatomy (lower/upper pole), duplication type, and severity of obstruction on a dynamic renogram. Although the most common problems of duplex systems are ureteroceles and reflux, UPJO should always be considered.

## Figures and Tables

**Table 1:** Characteristics of patients and management of UPJO associated with a duplicated collecting system.

Age	Gender	Type of duplication	Localization of duplication	Mean function of the involved renal moiety (%)	Type of surgery	Associated anomalies
4 months	Female	Incomplete	Right lower moiety	43	Ureteropyelostomy	
7 years	Male	Complete	Left lower moiety	54	Pyeloplasty	
6 years	Female	Complete	Right lower moiety	54	Pyeloplasty	
2 years	Male	Complete	Left lower moiety	34	Pyeloplasty	Vesicoureteral reflux
1.5 years	Female	Complete	Right upper moiety	3	Heminephrectomy	Right ureterovesical junction obstruction
			Right lower moiety	41	Ureteropyelostomy	
1 year	Male	Incomplete	Right renal lower moiety	42	Pyeloplasty	Right upper bagpipe renal collecting anomaly
			Right upper renal moiety	4	Heminephrectomy	Bilateral undescended testes
1 year	Male	Complete	Right renal upper moiety	0	Heminephrectomy	Left renal multicystic kidney
6 months	Male	Incomplete	Right lower moiety	35	Pyelopyelostomy	
1 year	Male	Complete	Left lower moiety	59	Pyeloplasty	Horseshoe kidney + penoscrotal hypospadias
1 year	Male	Complete	Left lower moiety	55	Pyeloplasty	
2 months	Female	Complete	Right lower moiety	19	Pyeloplasty	Right upper collecting system ends with ureterocele
3 months	Female	Complete	Right lower moiety	23	Pyeloplasty	
5 months	Male	Incomplete	Right lower moiety	45	Pyelopyelostomy	Right renal with triplex system
3 years	Male	Incomplete	Left lower moiety	21	Pyelopyelostomy	
1.5 years	Female	Incomplete	Left upper moiety	2	Heminephrectomy	
5 years	Male	Incomplete	Right lower moiety	34	Ureteropyelostomy	Vesicoureteral reflux
